# Identification of hub genes associated with RNAi-induced silencing of XIAP through targeted proteomics approach in MCF7 cells

**DOI:** 10.1186/s13578-020-00437-9

**Published:** 2020-06-11

**Authors:** Mehdi Agha Gholizadeh, Fatemeh T. Shamsabadi, Ahad Yamchi, Masoud Golalipour, Gagan Deep Jhingan, Majid Shahbazi

**Affiliations:** 1 Medical Cellular and Molecular Research Center, Golestan University of Medical Sciences, Zip Code: 4934174515, Gorgan, Iran; 2 Department of Biotechnology, Gorgan University of Agricultural Sciences and Natural Resources, Gorgan, Iran; 3 VProteomics, K-37A, Ground Floor Green Park Main, New Delhi, 110016 India; 4 AryaTinaGene Biopharmaceutical Company, Gorgan, Iran

**Keywords:** Breast cancer, XIAP, RNA interference, Apoptosis, Molecular targets, Proteomics

## Abstract

**Background:**

The X-linked inhibitor of apoptosis protein (XIAP) is the most potent caspase inhibitor of the IAP family in apoptosis pathway. This study aims to identify the molecular targets of XIAP in human breast cancer cells exposed to XIAP siRNA by proteomics screening. The expression of XIAP was reduced in MCF-7 breast cancer cells by siRNA. Cell viability and the mRNA expression level of this gene were evaluated by MTS and quantitative real-time PCR procedures, respectively. Subsequently, the XIAP protein level was visualized by Western blotting and analyzed by two-dimensional (2D) electrophoresis and LC–ESI–MS/MS.

**Results:**

Following XIAP silencing, cell proliferation was reduced in XIAP siRNA transfected cells. The mRNA transcription and protein expression of XIAP were decreased in cells exposed to XIAP siRNA than si-NEG. We identified 30 proteins that were regulated by XIAP, of which 27 down-regulated and 3 up-regulated. The most down-regulated proteins belonged to the Heat Shock Proteins family. They participate in cancer related processes including apoptosis and MAPK signaling pathway. Reduced expression of HSP90B1 was associated with apoptosis induction by androgen receptor and prostate specific antigen. Suppression of XIAP resulted in the enhancement of GDIB, ENO1, and CH60 proteins expression. The network analysis of XIAP-regulated proteins identified HSPA8, HSP90AA1, ENO1, and HSPA9 as key nodes in terms of degree and betweenness centrality methods.

**Conclusions:**

These results suggested that XIAP may have a number of biological functions in a diverse set of non-apoptotic signaling pathways and may provide an insight into the biomedical significance of XIAP over-expression in MCF-7 cells.

## Background

Inhibition of the apoptotic signaling pathway plays an important role in the initiation and progression of human cancers [1]. Various molecular mechanisms cause inhibition of apoptosis in cancer cells. High expression level of inhibitor apoptosis proteins (IAPs) is one of the important mechanisms that prevent apoptosis. IAPs are a family of anti-apoptotic proteins that are conserved throughout evolution from drosophila to vertebrates [2]. These proteins directly inhibit the terminal effector caspases through BIR-dependent recognition [3]. Over-expression of the IAPs occurs in many human tumor types that lead to cancer cell survival [4, 5]. In human, eight members of the IAP family have been identified. The most potent caspase inhibitor of this family is X-linked inhibitor of apoptosis (XIAP) [6]. XIAP inhibits the enzymatic activity of caspases at both the initiation phase and the execution phase by binding to caspases of 9, 3, and 7 [7]. High levels of XIAP was detected in human tumors and has been shown to confer the resistance to the chemotherapy drugs [8, 9]. Also, high throughput data analysis revealed that the expression of this gene remarkably increased in most of the cancers except ovary, kidney renal clear cells, and uterin cancers [10]. A logical strategy, therefore, to overcome the resistance to apoptosis is to target the factors that inhibit apoptosis pathway.

Our recent trends in the identification of regulated proteins by the critical genes in breast cancer cell progression such as PTTG1 and FOXO [11, 12], resulted in a proliferation of studies that have led to a renewed interest in XIAP. Accordingly, this study was designed to investigate the XIAP-regulated proteins by its suppression in MCF-7 cells. Specific down-regulation of XIAP by siRNA inhibited the growth of MCF-7 cells in vitro. We have also undertaken a proteomics pattern and pathway analysis to identify the biological targets of XIAP protein. XIAP suppression displayed enhanced expression of GDIB and CH60 along with metabolic gene encoding ENO1. The finding of this research revealed that XIAP may have a number of other biological functions in addition to participating in the mechanism of apoptosis, suggesting its potential value in tumor gene therapy.

## Materials and methods

### Materials

The human MCF-7 cells (breast carcinoma cell line) were obtained from Pasteur Institute of Iran (Tehran, Iran). Antibodies to XIAP; β-actin and the protease inhibitor cocktail were obtained from Sigma Co. (St. Louis, Mo). The XIAP siRNA was purchased from Santa Cruz Biotechnology Inc (sc-37508, Santa Cruz, Inc).

### Cell culture and transfection

The breast cancer cell line MCF-7 were cultured at 37 °C in 5% CO_2_ in RPMI-1640 medium (Invitrogen; Carlsbad, CA) containing 10% FBS (GIBCO) and 1% penicillin/streptomycin (Sigma, St. Louis, Mo). The cells were seeded in 6-well plates in 2 ml antibiotic-free normal growth medium supplemented with FBS (2 × 10^5^ cells/well). After incubation at 37 °C in 5% CO_2_ humidified atmosphere for 18 h, cells were transfected with 20 pmols XIAP siRNA through lipofection as described by the manufacturer. Then cells were incubated for 24, 48 and 72 h. Scrambled random siRNA (si-NEG) (Santa Cruz Biotechnology, Inc) was used as a control.

### Real-time PCR

Total RNA was extracted using TRIZOL reagent (Invitrogen, Italy) according to the manufacturer’s instructions. Samples were treated with RQ1 RNase-Free DNAse (Promega Corporation, USA) to eliminate possible contamination with genomic DNA. Strand cDNA synthesis performed with 1 μg DNase-treated RNA using the Transcriptor First Strand cDNA Synthesis Kit according to manufacturer’s instructions (Roche Applied Science). Real-time RT-PCR assay was performed on XIAP, ENO1, CH60, CRK, SAM50 and GAPDH genes using ABI 7300 real-time PCR systems (Applied Biosystems, Foster City, CA) thermal cycler detection system. The threshold cycle (Ct) of technical triplicates data were measured for each cDNA in the real-time PCR analysis. Each reaction consisting of 12.5 µl SYBR ^®^Green PCR Master Mix (Applied Biosystems, Foster City, CA), 1 µl cDNA (50 ng), 0.5 µl of each primer (10 pmol) and 10.5 µl nuclease-free water to conduct PCR in a 25 µl of a reaction mixture. The characteristics of primers were indicated in Table 1. We used the REST (Relative Expression Software Tool) software to analyze the reactive changes in gene expression. The number of specific transcripts was normalized to the expression level of GAPDH as the housekeeping gene.

**Table 1:** The sequence of applied oligonucleotide primers in the QRT-PCR experiment

Gene	Primer sequence (5′ to 3′)		Amplicon size
XIAP	F	ACCGTGCGGTGCTTTAGTT	133
	R	TGCGTGGCACTATTTTCAAGATA	
ENO1	F	CCTGCCCTGGTTAGCAAGAA	108
	R	GGCGTTCGCACCAAACTTAG	
CH60	F	GACGACCTGTCTCGCCG	78
	R	ATCTGGCGAAAGACTGTGGG	
CRK	F	AATCCGGGACAAGCCTGAAG	142
	R	ACCCTCCTGGTTACCTCCAA	
SAM50	F	ATGGAAGACCAGCCACACTG	128
	R	TGGCGTGCGAAAGAGATGAT	
GAPDH	F	GGTGGTCTCCTCTGACTTCAACA	121
	R	GTTGTAGCCAAATTCGTTGT	

“F” and “R” are abbreviation of Forward and Reverse primers

### Western blotting

Total protein was isolated by lysis buffer (contain 5% SDS, Triton X100, 100 mM Tris–HCl (PH = 4.7), sodium deoxycholate 150 mM NaCl, 5 mM EDTA, 10% glycerol) and 1% protease inhibitor Cocktail (Sigma-Aldrich). The protein concentration was quantified using Bradford assay. Protein levels were visualized by immunoblotting using antibodies against human XIAP (sc-11426, Santa Cruz Biotechnology, USA), and β-actin (Monoclonal Anti-β-actin, Sigma-Aldrich). Briefly, equivalent amounts of protein were resolved by SDS/polyacrylamide gel electrophoresis and transferred to a nitrocellulose membrane. For blocking, membrane incubated with 1X PBS, 0.1% Tween-20, 5% non-fat dry milk powder at 4 °C for 75 min. After washing with PBST, the membrane was incubated with anti-XIAP (1:800 dilution) and anti-β Actin (1:500 dilution) antibodies at 4 °C overnight. Subsequent washing the membrane with PBST for 5 times (1, 5, 10, 15, and 20 min), the membrane was incubated with the secondary antibody (goat Anti-Mouse IgG horseradish peroxidase-conjugated, Santa Cruz Biotechnology, USA) at a dilution of 1:500 for 2 h at room temperature. Protein bands were developed by Chemiluminescence using the Amersham ECL Detection Kit (ECL, ab65623, Abcam). Band densities were determined using NIH ImageJ software (US National Institutes of Health, Bethesda, MA, USA).

## 2D electrophoresis and image analysis

Total protein was quantified by 2-D quant kit (GE Healthcare) assay using BSA as standard. First dimension electrophoresis was performed with IPG strips (18 cm 3–10 NL, Immobiline Dry Strip; Bio-Rad) in IPGphor system (GE Healthcare) as previously described [13]. In brief, the rehydrated strips with 300 μL of loading buffer containing 1 mg of the protein sample were applied for the isoelectric focusing at 300 V for 2 h, 500 V for 2 h and 1000 V for 2 h. Then voltage ramped up to 8000 V in 5 h and continued at this voltage for a further 60,000 V. Focused strips were equilibrated for 15 min in reducing equilibration buffer (6 M urea, 50 mM Tris–HCl, pH 8.8, 30% v/v glycerol, 2% w/v SDS, a trace of bromo phenol blue and 1% w/v DTT) and for another 15 min in alkylating equilibration buffer with 2.5% w/v iodoacetamide instead of 1% DTT. The strips were transferred onto 12.5% SDS-PAGE gels and second dimension electrophoresis was performed at a constant current of 7.5 mA per gel for 30 min and 20 mA per gel for 5 h at 20 °C. Gels were fixed for 1 h in 40% methanol 10% acetic acid, washed for 30 min in ultrapure water and then stained with colloidal Coomassie Brilliant Blue G-250 [6]. Stained gels were scanned with Scanner Perfection (GE Healthcare) and image analysis, spot detection and spot pairing carried out using Image Master 2D Platinum 6.0 (GE Healthcare). Scatter plots between gels were used to estimate gel similarity (Image Master 2D Platinum 6.0 user manual). For selected protein spots, the corresponding relative spot intensity of three different experiments was measured. The induction ratios of each selected protein spot were determined by comparing in-gel intensities of XIAP siRNA and si-NEG transfected. These proteins-coding genes were introduced as the differentially expressed genes (DEGs).

### Protein identification by LC–ESI–MS/MS

The spots of protein were manually excised from the gels and completely destained with a 1:1 (v/v) solution of acetonitrile and 25 mM ammonium bicarbonate. The destained pieces were equilibrated in 5 mM ammonium bicarbonate, dried by vacuum centrifugation and finally digested in 5 mM ammonium bicarbonate supplemented by trypsin (10 ng/μl, sequencing grade modified trypsin, Promega) for at least 16 h at 37 °C. Digestion was stopped by addition of 1 μl of 10% trifluoroacetic acid (TFA) and supernatants collected. Gel pieces were extracted with 50 μL of a 1:1 (v/v) solution of acetonitrile and 0.1% TFA. Supernatants collected from the same samples pooled and dried by vacuum centrifugation. Samples were dissolved in 10 μl of 2% acetonitrile, 0.1% TFA. All the analysis was performed using a Q-TRAP (LC packings and Applied Biosystems/MDSSciex) LC–MS/MS system as described by Boudart et al. [7]. Mass data collected during analysis was processed by the Analyst software (Applied Biosystems/MDS Sciex) and the MS/MS lists used to search the Swiss-Prot database. Data lists blasted against the databases using the Mascot software (2.2.3). The search strategy was carried out according to some inclusion criteria including up to two missed cleavages, 0.5 Mass accuracy (for parent and the fragment ions), and variable modifications set for deamidation of arginine or glutamine and oxidation of methionine. Probability-based MASCOT scores used to evaluate the protein identifications. We used the MFPaQ program [14] version 4 to validate the data.

### Cell proliferation assay

Cell viability was determined by MTS dye-reduction assay according to the provided instruction (Promega). The cells were seeded at a density of 2 × 10^3^ cells/well in 96-well plates cultured in media containing 100 μl of normal growth medium supplemented with 5% FBS. Subsequently, the viability of cells was evaluated by MTS reagent at 0, 24 and 48 h after transfection. Absorbance was determined by the BioTek Plate Reader at 490 nm. All assays were performed in triplicate and three times.

### System biology analysis of the regulated proteins by XIAP

In order to identify the regulated proteins by XIAP in breast cancer cells, the classification of differentially expressed proteins (DEPs) was performed by some bioinformatics databases. The STRING (v 11.0) database was applied for the interaction of XIAP with other DEPs. The included criteria for the selection of the partner of these proteins was the confidence with at least medium (score 0.4) and Kmeans clustering constructed according to the experimental, textmining, co-expression, and database knowledge. Moreover, gene ontology (GO) enrichment analysis and significant pathways were assessed by PANTHER (v 14.0) and KEGG resources, respectively.

In order to identify the hub genes in the protein–protein interaction network, network analyzer tool in Cytoscape software was applied. Then, the protein interaction relationship network was visualized based on the degree parameter.

### Statistical analysis

Statistical analysis was carried out using the SPSS version 18.0 software (SPSS Inc., Chicago, IL). Data were presented as mean value ± SD. Statistical analysis of the data was performed by the Student *t* test or the Mann–Whitney U test with the SigmaStat 3.0 software (SPSS). For all tests, a *P*-value ≤ 0.05 was considered as statistically significant.

## Results

### Declining XIAP mRNA and protein expression by XIAP siRNA

**Fig. 1:**
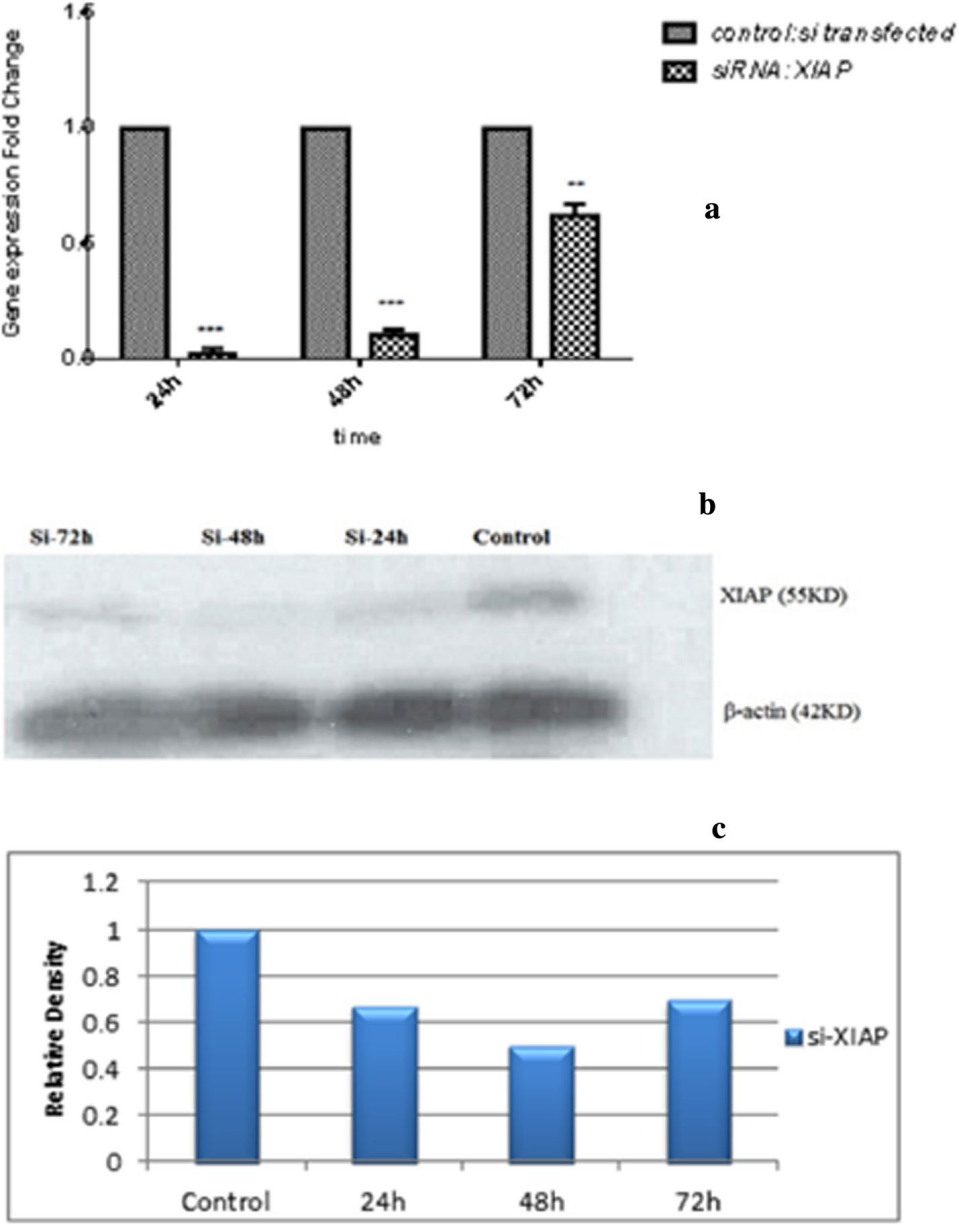
Expression of XIAP in response to XIAP silencing in MCF-7 cells. **a** Fold-change expression of XIAP gene. The expression level of XIAP mRNA was reduced in siRNA treated cells compared to the si-NEG after 24, 48 and 72 h post-transfection. ** illustrates the *P* < 0.001; *** represents *P* < 0.0001. **b** Protein expression level of XIAP in response to XIAP siRNA. Western blot shows that the highest down-regulation of protein occurred in 48 h post-transfection. **c** Densitometry of XIAP protein bands. The results of western blot were quantified by ImageJ software in order to measure the density of protein bands. The si-NEG was employed as a control group which exhibits the normal expression quantity of XIAP. The densitometry analysis revealed the low amount of XIAP protein at 48 h after transfection

The *XIAP* mRNA was found to be successfully down-regulated at 24, 48 and 72 h post-transfection (Fig. 1a). As can be seen from this Figure, the expression of XIAP was significantly reduced at all times, especially at 24 and 48 h (*P *< 0.0001).

The protein expression of XIAP in response to the siRNA against XIAP in MCF-7 cells was reduced at 24, 48 and 72 h (Fig. 1b). To semi-quantify the amount of XIAP protein and evaluate the efficient acting time of siRNA, the density of protein bands was measured. Figure 1c shows the highest reduction in *XIAP* protein level occurred at 48 h after siRNA transfection.

### XIAP siRNA inhibits the MCF-7 proliferation

**Fig. 2:**
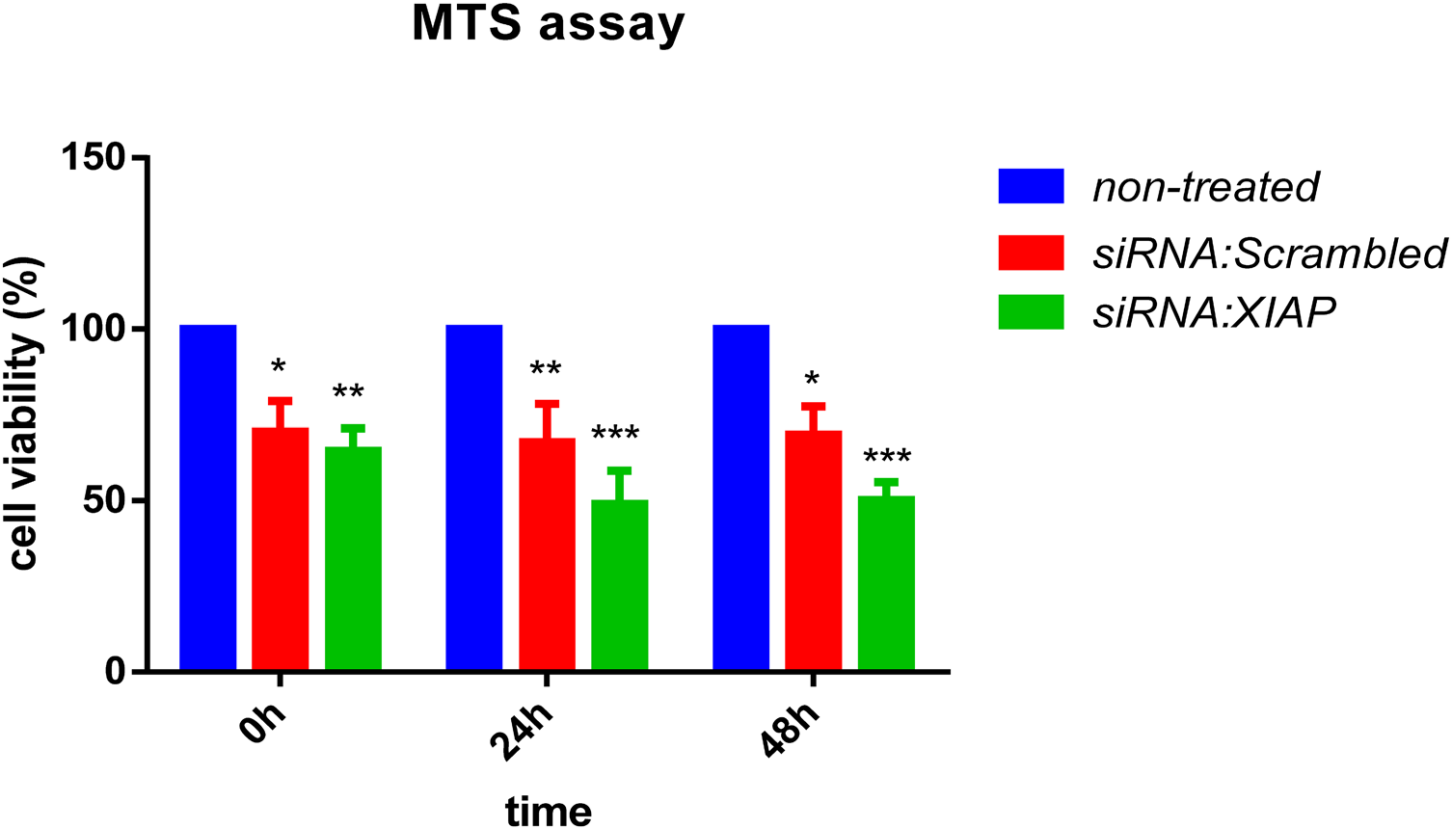
Suppression of MCF-7 cell proliferation in post-transfection of siRNA XIAP. The highest reduction of cell viability was observed at 24 and 48 h post-transfection. Single star represent P < 0.05, ** shows *P* < 0.001, *** *P* < 0.0001

To distinguish the role of XIAP in the proliferation of breast cancer cells, MTS assay was carried out at 0, 24, and 48 h subsequent of silencing (Fig. 2). Cell growth was significantly reduced in XIAP siRNA treated cells compared to the scrambled siRNA treated and non-treated cells (*P *< 0.05).

### XIAP silencing-regulated proteins

**Fig. 3:**
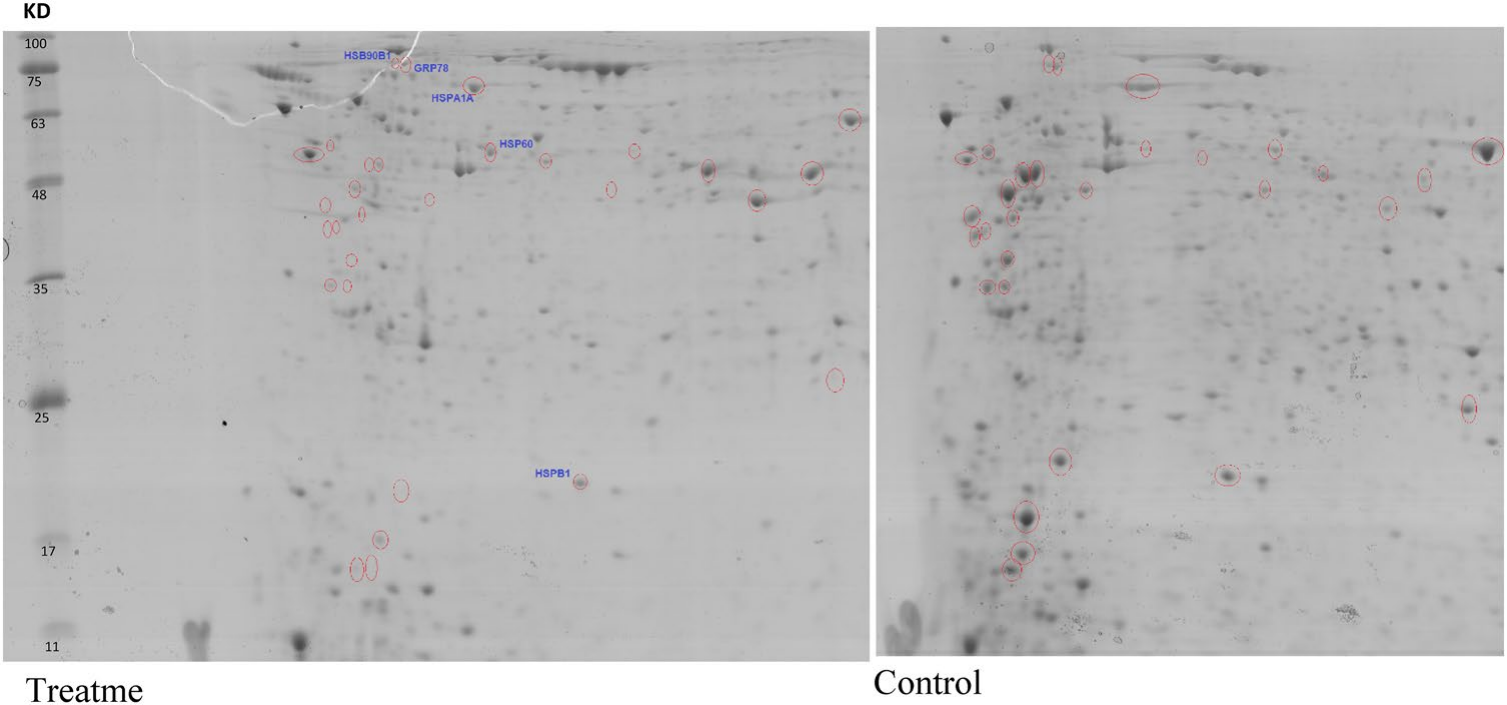
Two-dimensional gel electrophoresis of MCF-7 cells exposed to the XIAP siRNA. After silencing of XIAP, the expression of 30 proteins was differentiated in the XIAP siRNA-transfected cells compared to the si-NEG. The non-linear 18 cm, 3 to 10 pH range IPG strips in the first dimension and 12.5% polyacrylamide gel in the second dimension were used

According to the first set of statistical analysis, the differences in the proteins expression which are regulated by XIAP were identified at 48 h post-transfection due to the low expression level of XIAP protein at this time. Following 2D gel analysis, approximately 1000 well-resolved spots were detected in each gel (Fig. 3). Then, the nature of DEPs were assessed by the mass spectrometry. The Uniprot entry of the DEPs along with their main characteristics were presented in Table 2. It is apparent from this table that the expression of 30 proteins are altered in response to the XIAP silencing than si-NEG treated cells, of which 27 down-regulated and 3 up-regulated. The scores of theoretical mass and isoelectric point were compared in order to the accuracy of the identified DEPs. The result indicates that these scores are nearly the same, suggesting that they could be closely located on 2D-PAGE.

**Table 2:** Differentially expressed proteins in response to siRNA against XIAP in MCF-7 cells

Spot	Gene product	Score	Uniprot ID	Biological process	Molecular function	Theoretical MW (KD)/pI	On gel MW (KD)/pI	Location	Ratio^*^
1	GRP78	286	P11021	Protein metabolism	Chaperone activity	72,402/5.07	77,112/4.65	9q33	0.33
2	ENPL	82	P14625	Protein metabolism	Heat shock protein activity	92,696/4.76	80,516/4.55	12q23	0.27
3	ATPB	390	P06576	Metabolism, Energy pathways	Transporter activity	56,525/5.26	52,427/4.65	12q13	0.66
4	GDIB	103	P50395	Transport	Auxiliary transport protein activity	51,087/6.11	46,950/6.1	10p15	2.3
5	GRP75	274	P38646	Protein metabolism	Chaperone activity	73,920/5.87	71,587/5.9	5q31	0.51
6	G6PD	257	P11413	Metabolism, Energy pathways	Catalytic activity	59,675/6.39	55,639/6.1	Xq28	0.49
7	ENO1	735	P06733	Metabolism, Energy pathways	Catalytic activity	47,481/7.01	45,701/6.3	1p36	3.3
8	ALBU	73	P02768	Transport	Transporter activity	71,317/5.92	66,546/5.4	4q13	0.51
9	PRDX2	401	P32119	Metabolism; Energy pathways	Peroxidase activity	22,049/5.66	22,089/5.4	19p13	0.39
10	TCPE	122	P48643	Protein metabolism	Chaperone activity	60,089/5.45	64,545/5.3	5p15	0.64
11	FKBP4	102	Q02790	Metabolism, Energy pathways	Isomerase activity	52,057/5.35	50,535/5.2	12p13	0.43
12	HS71A	234	P0DMV8	Protein metabolism	Chaperone activity	70,294/5.48	71,325/5.3	6p21	0.68
13	HS71B	234	P0DMV9	Protein metabolism	Chaperone activity	70,294/5.48	81,261/5.3	6p21	0.41
14	HSP7C	526	P11142	Protein metabolism	Heat shock protein activity	71,082/5.37	71,261/5.4	11q24.1	0.68
15	HS90A	32	P07900	Protein metabolism	Chaperone activity	85,006/4.94	91,559/4.7	14q32	0.1
16	NPM	109	P06748	Protein metabolism	Chaperone activity	32,726/4.64	38,294/4.3	5q35	0.65
17	CRK	103	P46108	Cell communication, Signal transduction	Receptor signaling complex scaffold activity	338,675.38	29,195/5.0	17p13	0.03
18	PA2G4	70	Q9UQ80	Regulation of nucleobase, nucleoside, nucleotide and nucleic acid metabolism	Transcription regulator activity	44,101/6.13	70,963/6.3	12q13	0.63
19	SERA	140	O43175	Metabolism; Energy pathways	Catalytic activity	57,356/6.29	77,142/5.4	1p12	2.72
20	TBA1B	840	P68363	Cell growth and/or maintenance	Structural molecule activity	50,804/4.94	58,606/4.6	12q13	0.65
21	SAM50	36	Q9Y512	unknown	unknown	52,342/6.44	17,144/5.4	22q13	0.07
22	KPYM	118	P14618	Energy pathways, metabolism	Kinase activity	58,470/7.96	60,963/6.3	15q23	0.63
23	PRDX6	112	P30041	Metabolism, Energy pathways	Peroxidase activity	25,133/6.00	27,314/5.9	1q25	0.06
24	HSPB1	414	P04792	Protein metabolism	Chaperone activity	22,826/5.98	20,312/5.7	7q11	0.41
25	CH60	623	P10809	Protein metabolism	Heat shock protein activity	61,187/5.70	68,904/5.4	2q33	2.3
26	TCPZ	93	P40227	Protein metabolism	Chaperone activity	58,444/6.23	60,904/6.0	7p11	0.44
27	TBB5	504	P07437	Cell growth and/or maintenance	Structural constituent of cytoskeleton	50,095/4.78	54,486/4.8	6p21	0.75
28	IF5A1	90	P63241	Protein metabolism	Translation factor activity, nucleic acid binding	17,049/5.08	1646/4.8	17p13	0.6
29	EF1G	148	P26641	Protein metabolism	Translation regulator activity	50,429/6.25	53,625/5.9	11q12	0.5
30	PDIA1	635	P07237	Protein metabolism	Isomerase activity	57,480/4.76	60,784/4.5	17q25	0.83

The product of CH60, ENO1, and GDIB genes were increasingly expressed (> twofold)

Furthermore, to verify the proteomics results, the level of four proteins was assessed by the quantitative real-time PCR. The expression level of these genes confirmed the 2D-PAGE results (Table 3). The transcript expression fold of ENO1, CH60, CRK and SAM50 genes were 4.63, 5.81, 0.25 and 0.4, respectively.

**Table 3:** Transcripts expression corresponding to the differentially expressed proteins in response to XIAP silencing

Uniprot	Entry name	Protein name	Transcriptomics	Proteomics
P06733	ENO1	Alpha-enolase	4.63	3.3
P10809	CH60	60 kDa heat shock protein, mitochondrial	5.81	2.3
Q9Y512	SAM50	Sorting and assembly machinery component 50 homolog	0.4	0.07
P46108	CRK	Adapter molecule crk	0.25	0.03

### Protein interactions and network analysis

**Fig. 4:**
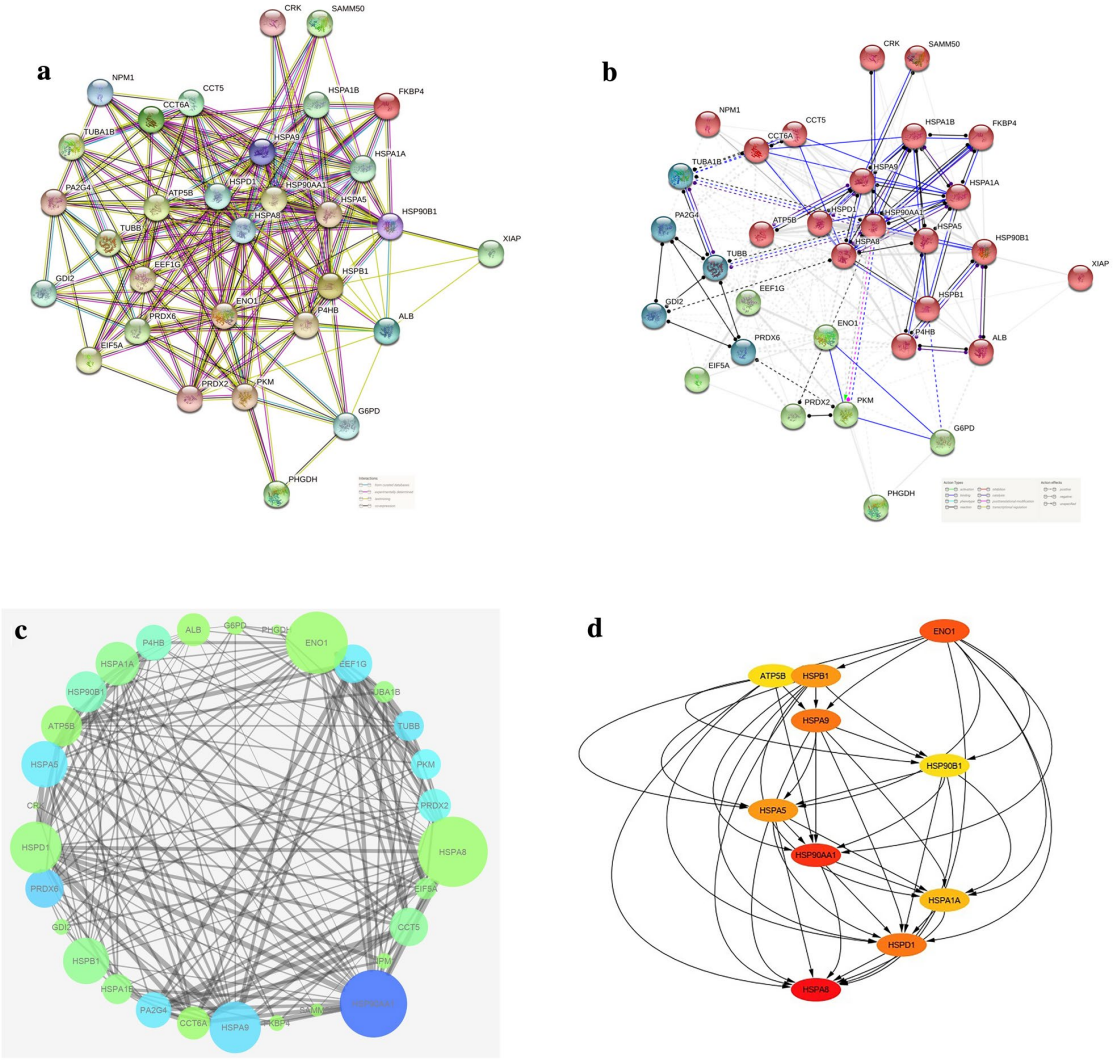
Functional classification of DEPs in MCF-7 cells exposed to RNAi-induced silencing of XIAP. **a** A network of protein–protein interaction among the XIAP-regulated proteins was constructed. XIAP did not directly interact with the identified DEPs in MCF-7 cells. According to the textmining criteria, XIAP interacts with HSP90B1, HSPB1, and ALB. **b** The molecular function of the XIAP-regulated proteins are demonstrated. The precise mechanism of action of XIAP with the DEPs was not identified yet. Colors and shapes of line show binding (blue), reaction (black), catalysis (indigo), activation (light green), post-translational modification (fuchsia). The positive and negative effects were illustrated by arrowhead and bar, respectively. **c** Network of regulated proteins by XIAP was constructed according to the degree, betweenness centrality, and co-expression by CytoHubba application in Cytoscape software. The thickness of grey lines indicates the strength of co-expression. HSP90AA1 and HSPA6 were identified as the hub genes in this network. Although, it should not be ignored the impact of HSPA8 and ENO1 genes

The association and interaction of XIAP with other DEPs was analyzed by STRING. Figure 4a illustrates a complex network of XIAP-regulated proteins. From this data, it can be seen that XIAP did not directly interact with the identified DEPs in MCF-7 cells exposed to the XIAP siRNA. According to the STRING results, we found that XIAP interacts with Heat shock proteins (HSPs), HSP90B1 and HSPB1, and ALB based on the textmining criteria with minimum confidence. To assess the function of regulated proteins by XIAP, reconstruct a network by STRING molecular action (Fig. 4b). The molecular mechanism and type of interaction of XIAP with the DEPs were not realized.

The application of CytoHubba was used to identify the key nodes in the mentioned network. Figure 4c depicts a network of regulated proteins by XIAP according to the degree, betweenness centrality, and co-expression methods. The finding of this analysis indicated that HSP90AA1 and HSPA9 are key nodes in this network based on the degree method. While in terms of betweenness centrality, HSPA8 and ENO1 are critical nodes. Additionally, ENO1 gene activated other top 10 genes, and HSPA8 along with HSPD1 were regulated by others (Fig. 4d).

### Gene ontology enrichment analysis

**Fig. 5:**
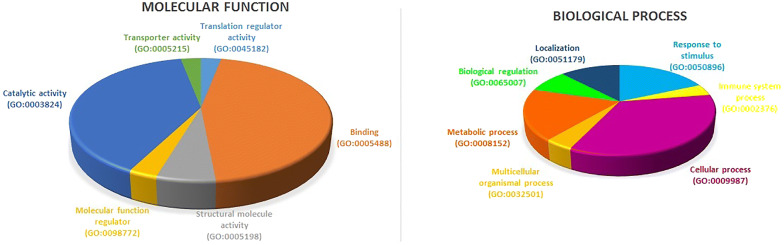
GO analysis of the XIAP-regulated proteins. The number of genes involved in molecular function (**a**) and biological process (**b**) were demonstrated. Enrichment analysis revealed that most of regulated proteins by XIAP have binding and catalytic activities. Also, these proteins are implicated in cellular and metabolic processes

The number of genes involved in each class of biological process and molecular function was shown in Fig. 5. The GO enrichment analysis demonstrated that proteins regulated by XIAP are mostly involved in cellular and metabolic processes along with response to stimulus. Accordingly, these results suggested a critical role of XIAP in the action of these pathways.

**Fig. 6:**
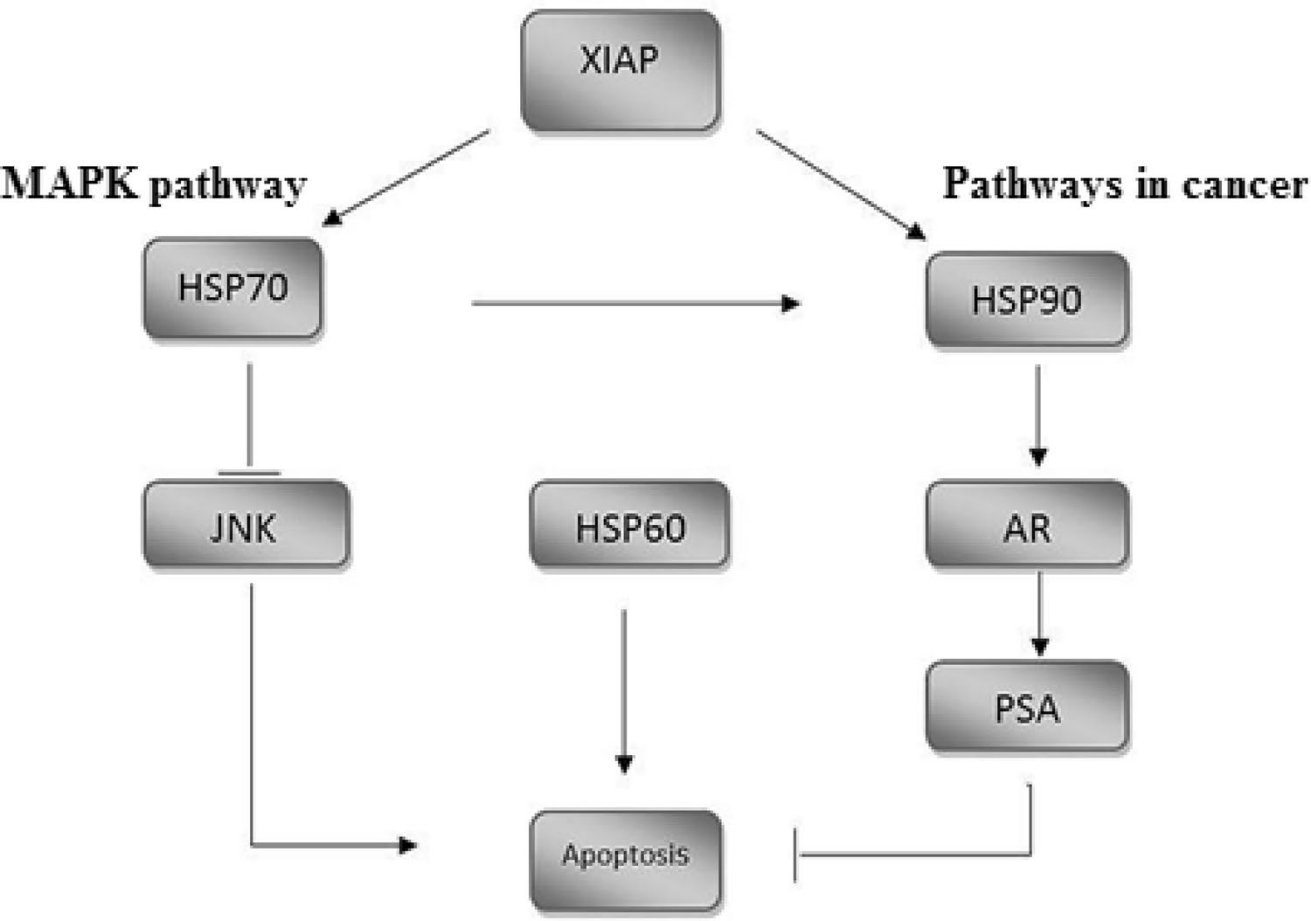
Proposed pathway for activation of apoptosis after XIAP silencing. Association of XIAP in the MAP-kinase signaling pathway is demonstrated in cancer

Further analysis by KEGG pathway bioinformatics database showed that the involvement of XIAP molecular targets in various biological pathways. HSPs had a critical role in inhibition of apoptosis in cancer pathway and MAPK signaling pathway. Cancer signaling pathways (hsa05200) showed that HSP90B1 promotes cell proliferation and evades apoptosis by activating of androgen receptor (AR) and PSA. Also, activation of PSA causes cell proliferation survival in prostate cancer signaling pathway. In MAPK signaling pathway (hsa04010), HSPA1A interacts with Evil and suppresses apoptosis by the inhibition of JNK. These results suggested that XIAP may plays important roles in a diverse set of non-apoptotic signaling pathway in breast cancer and may have potential value in tumor gene therapy (Fig. 6).

## Discussion

The main obstacle of cancer therapy is the identification of specific factor of tumor cells. Inhibitor of apoptosis proteins especially XIAP are one of the main influencing agents in carcinogenesis. The XIAP transcript has been frequently expressed in different cancers [10]. Interestingly, its expression in both triple positive (MCF-7) and triple negative (MDA-MB-231) breast cancer cells were equal and higher than the non-tumorigenic mammary epithelial cells (MCF-10), have noted the impact of XIAP in proliferation of breast tumor cells. Nevertheless, very little was found on the XIAP functional mechanism in breast cancer. Consequently, the present study was aimed to identify the protein targets regulated by XIAP in the proliferation of cancer cells in MCF-7 cell line.

Our data showed that down-regulation of XIAP resulted in the reduction of cell proliferation and changed the expression pattern of 30 proteins in MCF-7 cells. The interesting point of proliferation assay was that the most remarkable reduction in cell proliferation occurred at 48 h post-transfection (*P *< 0.0001); at the same time period that the expression of XIAP reduced. These findings further confirm the idea of XIAP involvement into the signaling pathways relevant to the oncogenesis or progression of the MCF-7 cells through the enrichment analysis of DEPs (Fig. 5).

By silencing of XIAP, ENO1 (+3.3) was the first highly expressed protein. From the oncology point of view, this protein acts in cell progression, apoptosis, and tumor cell invasion processes through repression of MYC gene [15] and activation of FAK in the PI3K/AKT pathway [16]. It introduced as an oncogene-associated protein in hepatocellular carcinoma [17]. Our research team also revealed the association of enhanced expression of ENO1 with PTTG1 [11]. Likewise, this protein participates in glycolysis and HIF1 signaling pathways to promote anaerobic metabolism (hsa04066). Interestingly, our network analysis displayed the impact of ENO1 as an activator of the top 10 genes.

The last two up-regulated proteins were GDIB (P50395) and CH60 (P10809) with 2.3 fold change expression ratio. The increased transcription of both genes were indicated in breast invasive carcinoma [10]. GDP dissociation inhibitor 2 was introduced as a suppressor of tumor metastasis and invasion [18]. It has been shown that low expression of GDIB is associated with increased metastasis risk and decrease survival in patients harboring bladder cancer. Also, other researcher demonstrated that XIAP silencing resulted in the decline of GDIB expression accompanied with reduced cell invasion in bladder cancer [19]. Although, our current findings do not support the earlier research. This inconsistency may be due to the different cancer types.

CH60 protein, a typical mitochondrial chaperone with tumor suppressor activity, is participated in the cancer cell progression. It has been localized in the ER during breast cancer cell apoptosis [20]. It was reported that HSP60 silencing considerably increased the migration and invasion phenotypes in the head and neck cancer cells [21]. They mentioned that deficiency of Hsp60 tumor suppression function contributed to the aggressive cancers. As a result, down-regulation of XIAP may cause the up-regulation of HSP60 which could be resulted in the activation of apoptosis and reduced the proliferation of cells (Fig. 6).

We found that the HSPs production (# 12) reduced by the treatment of XIAP siRNA except HSP60. Their functional mechanism was demonstrated in post-translational regulation of signaling molecules, protein folding, and inhibition of apoptosis under stressful situation [22, 23]. High expression of these molecules has been reported in various cancers, suggesting their contribution to the survival of tumor cells [10, 24]. They are crucial for the maintenance of cell integrity during both normal cell growth and pathophysiological condition [25, 26]. The involvement of HSPs in tumor development were indicated with distinct immunologic mechanisms promoting cell growth [27, 28]. These molecules have an important role in the apoptosis inhibition and promotion of cell proliferation in different signaling pathways including MAPK signaling and pathways in cancer (Fig. 6).

The observed finding in this study corroborated the results of a wide number of previous works that have examined the activity of HSPs in cancer related pathways [22]. For instance, HSP70 is the most central family of stress proteins that recently empirical evidence introduced XIAP as a client of Hsp70 in MDA-MB-231 breast cancer cells [29]. Our proteomics results showed that expression of HSP70 has reduced after silencing of XIAP gene. It has been exhibited that the resistance to apoptosis in some tumors is due to the binding between HSP70 and the apoptosis protease activating factor (Apaf-1) gene [30]. Also, these findings are in line with a prior study that revealed multiple HSP70-binding sites on XIAP [31]. It can then be assumed that the XIAP protein participates in apoptosis by affecting the expression of HSP70 through the MAPK-signaling pathway (Fig. 6).

Moreover, we found that the expression of HSP90 isoforms has reduced after XIAP silencing. HSP90 is a molecular chaperone that is up-regulated in various cancer types and required for the folding of numerous signaling proteins.

In this study, silencing of XIAP attenuated the expression of glucose-regulated protein, GRP78. It is an endoplasmic reticulum (ER) chaperone initially discovered as proteins inducible by glucose starvation [32]. Although GRP78 is predominantly found within the ER lumen, recent studies have shown that it can redistribute, under ER stress condition, tumor cell surface and mitochondria [33, 34]. Li N, Zoubeidi et al. showed that under ER stress, GRP78 and Clusterin function cooperatively mediated the anti-apoptotic effects in the mitochondria pathway [35]. GRP94 is the resident HSP90 molecular chaperone that modulates protein folding, processing, and secretion. Our data showed that HSP90B1 have a critical role in cancer signaling pathway. In this pathway, HSP90B1 promotes the proliferation of cancer cells and their resistance to apoptosis by activating of PSA (Fig. 6). It has also been reported that HSP90B1 helps cells to escape apoptosis and influence the function of various proto-oncogenes that are essential for breast cancer growth [26]. The role of this protein in prostate cancer cell proliferation was demonstrated by activating androgen receptor (AR). The interaction between AR and the promoter region of HSPA1B in this cell line was resulted in the regulation of HSP70 [36].

It is possible, therefore, that XIAP silencing could be a major factor, if not the only one, causing inhibition of cell progression and apoptosis induction by reduction of HSPs expression such as HSP90 and HSP90B1. However, this is an imperative issue for further research to explore on the molecular action of XIAP in apoptosis induction through HSPs members.

## Conclusion

In conclusion, our results showed that XIAP protein may have diverse role in the biological signaling pathways by altering expression profile of other proteins especially, HSPs family. The present study provides additional evidence on the XAIP-mediated inhibition of apoptosis and enhancement of cell proliferation in breast cancer in vitro. These findings will almost certainly be much perused and scrutinized. However, in further research, it might be possible to determine the regulated proteins by XIAP in different breast cancer cell lines. Indeed, more investigation on this topic necessitates to be undertaken before the association between XIAP and breast cancer is more clearly understood. Notwithstanding these limitations, the study offers some insight into the type of interactions between XIAP and its regulated proteins.

## Data Availability

The datasets used and/or analyzed during the current study are available from the corresponding author on reasonable request.
